# Neonatal Maternal Separation Induces Sexual Dimorphism in Brain Development: The Influence on Amino Acid Levels and Cognitive Disorders

**DOI:** 10.3390/biom13101449

**Published:** 2023-09-26

**Authors:** Jolanta H. Kotlinska, Pawel Grochecki, Agnieszka Michalak, Anna Pankowska, Katarzyna Kochalska, Piotr Suder, Joanna Ner-Kluza, Dariusz Matosiuk, Marta Marszalek-Grabska

**Affiliations:** 1 Department of Pharmacology and Pharmacodynamics, Medical University, Chodzki 4A, 20-093 Lublin, Poland; pawel.grochecki@umlub.pl; 2 Independent Laboratory of Behavioral Studies, Medical University, Chodzki 4A, 20-093 Lublin, Poland; agnieszka.michalak@umlub.pl; 3 Department of Radiography, Medical University, Staszica 16, 20-081 Lublin, Poland; anna.pankowska@umlub.pl (A.P.); katarzyna.kochalska@umlub.pl (K.K.); 4 Department of Analytical Chemistry and Biochemistry, Faculty of Materials Science and Ceramics, AGH University of Science and Technology, A. Mickiewicza 30, 30-059 Krakow, Poland; piotr.suder@agh.edu.pl (P.S.); nerkluza@agh.edu.pl (J.N.-K.); 5 Department of Synthesis and Chemical Technology of Pharmaceutical Substances with Computer Modelling Lab, Medical University, Chodzki 4A, 20-093 Lublin, Poland; dariusz.matosiuk@umlub.pl; 6 Department of Experimental and Clinical Pharmacology, Medical University, Jaczewskiego 8B, 20-090 Lublin, Poland; marta.marszalek-grabska@umlub.pl

**Keywords:** maternal separation, recognition memory, hippocampus, amino acids, mGlu5, sex, rats

## Abstract

Repeated maternal separation (MS) is a useful experimental model in rodents for studying the long-term influence of early-life stress on brain neurophysiology. In our work, we assessed the effect of repeated MS (postnatal day (PND)1–21, 180 min/day) on the postnatal development of rat brain regions involved in memory using proton magnetic resonance spectroscopy (^1^HMRS) for tissue volume and the level of amino acids such as glutamate, aspartate, glutamine, glycine and gamma-aminobutyric acid (GABA) in the hippocampus. We assessed whether these effects are sex dependent. We also use novel object recognition (NOR) task to examine the effect of MS on memory and the effect of ethanol on it. Finally, we attempted to ameliorate postnatal stress-induced memory deficits by using VU-29, a positive allosteric modulator (PAM) of the metabotropic glutamate type 5 (mGlu5) receptor. In males, we noted deficits in the levels of glutamate, glycine and glutamine and increases in GABA in the hippocampus. In addition, the values of perirhinal cortex, prefrontal cortex and insular cortex and CA3 were decreased in these animals. MS females, in contrast, demonstrated significant increase in glutamate levels and decrease in GABA levels in the hippocampus. Here, the CA1 values alone were increased. VU-29 administration ameliorated these cognitive deficits. Thus, MS stress disturbs amino acids levels mainly in the hippocampus of adult male rats, and enhancement of glutamate neurotransmission reversed recognition memory deficits in these animals.

## 1. Introduction

Early life is a critical period for brain development, both in humans and rodents, during which neuronal plasticity, synaptic organization and remodelling activities rapidly develop [1]. Stress experienced early in life has deleterious impact on postnatal brain development and affects functional maturation of critical brain regions, including the hippocampus, which is responsible for cognitive and affective functions [2,3,4,5,6]. Thus, early life stress is an important risk factor for several forms of cognitive decline at a later age [7,8], including Alzheimer’s Disease [9,10]. However, the neurobiological substrates of stress-associated effects on cognitive function, and the sex bias in risk for developing stress-associated pathology, are poorly understood.

MS is an animal model that resembles the stress of early life negative experiences. It is considered an analogue of childhood abuse or mistreatment [11]. In this animal model, the forced absence of the dam produces alterations in neuroendocrine, cognitive and behavioural functioning, as well as plastic changes in the offsprings’ brains that persist into adulthood [12,13,14,15]. In the MS protocol, pups are separated daily from their dam, usually from postnatal day (PND)1 to 14 or (PND)1–21, for short or long periods [16,17]. Prolonged periods of MS (180 min) have been shown to be an intense stressor for the litter, and can negatively impact maternal care during behavioural, physiological and neuroendocrine maturation [18]. Several lines of evidence suggest that MS modifies the activity of different neurotransmitters responsible for the cognitive/memory deficits in adult animals [19,20], including glutamate [21,22].

Glutamate (Glu) is the most abundant amino acid in the brain and the major excitatory neurotransmitter in the mammalian central nervous system (CNS) [23]. Glu is an agonist of ionotropic receptors (N-methyl-D-aspartate (NMDA), α-amino-3-hydroxyl-5-methyl-4-isoxazole-propionate (AMPA), kainate) that are the ligand-gated ion channels that mediate fast excitatory synaptic transmission, as well as the family of metabotropic glutamate (mGlu1–8) receptors that belong to the G-protein-coupled receptors (GPCRs) and modulate cascades of intracellular second messengers [24]. Under physiological conditions, Glu is especially important for proper neuronal development, synaptic plasticity, learning and memory [23]. Throughout development, a connecting mechanism may exist that links glutamatergic NMDA receptor activation to changes in dendritic morphology, and affect connectivity [25,26].

A tight control of extracellular Glu level is crucial for the correct functioning and development of central glutamatergic synapses and neural circuits. Glycine, the simplest amino acid, acts as a cofactor required for activation of the NMDA receptors [27], but another amino acid, glutamine (Gln) (from the astrocytes), is the predominant source of glutamate in glutamatergic terminals [28,29]. There is increasing evidence that Gln and Glu are essential amino acids that play important roles in maintaining growth and health in both neonates and adults [30]. Furthermore, the activity of glutamatergic excitatory neurons is coordinated by an intricate network of inhibitory gamma-aminobutyric acid (GABA)ergic interneurons [31]. The balance between excitatory and inhibitory synaptic transmission is essential to ensure proper information processing and in maintaining a finely tuned balance in neural activity, which is vital for central physiological functions [32,33].

Recognition memory is one of the first cognitive abilities maturating during juvenile development. Published data suggest that this memory relies on a neuronal network that includes the prefrontal cortex, hippocampus and perirhinal cortex [34,35,36]. Although the role of the hippocampus in nonspatial object memory remains highly debated due to conflicting findings [36,37,38,39], early-life stress can lead to progressive impairments of hippocampal function and cause decline in recognition memory in adult animals [40,41]. The NOR task is a commonly used test to evaluate recognition memory in rodents. In this task, subjects must spontaneously explore a pair of identical objects and, after a delay, distinguish between the now familiar objects and novel objects [42]. It has been shown that MS affects recognition memory [43,44,45,46] and induces changes in the hippocampal glutamate neurotransmission in adult rats [21,47]. The NOR task is a simple method that does not need external stimulus (e.g., reward or punishment), but only requires a little training or habituation. This task can be performed in a short time so animals do not feel stressed, and it can examine the recognition memory after only one trial, which gives it an advantage over other, more complex, methods [48].

In our study, we assessed the impact of long-term repeated MS ((PND)1–21, 180 min/day) on the postnatal development of rat brain regions involved in memory by using proton magnetic resonance spectroscopy (^1^HMRS) to assess tissue volume and the changes in the level of glutamate and related amino acids such as aspartate, Gln, glycine and GABA in the hippocampus. We also determined whether these effects are sex dependent. Moreover, we employed the NOR task to examine the effect of MS on the memory of adult rats ((PND)60). Furthermore, because ethanol affects glutamatergic neurotransmission [49], and rodents with MS exhibit enhanced vulnerability to the ethanol intake [50], we evaluated the impact of acute ethanol administration (at a dose that did not cause memory loss or memory impairment) on recognition memory deficits in MS male rats. Subsequently, we attempted to ameliorate postnatal stress-induced memory deficits by using VU-29, a positive allosteric modulator (PAM) of the mGlu type 5 (mGlu5) receptor. Activation of, particularly, the mGlu5 receptor, has a critical role in induction of NMDA-receptor-dependent forms of synaptic plasticity and excitotoxicity [51,52].

## 2. Material and Methods

### 2.1. Animals

Approval of this study gained from the Local Ethics Committee (79/2021) in Lublin under the “3R approach” (Replace, Reduce and Refine). It was also performed according to the National Institute of Health Guidelines for the Care and Use of Laboratory Animals and The European Community Council Directive of November 2010 for Care and Use of Laboratory Animals (Directive, 2010/63/EU) (IACUC equivalent approval). The offspring of Wistar dams (OMD, Lublin, Poland) were the subjects of the experiment. During the gestation period, the dams were housed individually in polypropylene cages (41 × 34 × 16 cm) with the cage floor covered with an approximately 3 cm layer of sawdust shavings. Throughout the study, rodent chow (Sniff Specialization GmbH, Sorest, Germany) and water were available at libitum. All experiments were carried out between 9:00 a.m. and 7:00 p.m. under standard laboratory conditions (22 ± 1 °C, 12:12 light/dark cycle, lights on at 8:00). The day of birth was designated at (PND)0.

### 2.2. Drugs

Ethanol (95%, *w*/*v*, Polmos, Poznan, Poland) was diluted in saline (0.9% NaCl) to a concentration of 10% (*w*/*v*) and administered at the dose of 1.5 g/kg, intraperitoneally (i.p.). This dose did not affect recognition memory in our preliminary study. The selective mGlu5 PAM N-(1,3-diphenyl-1H-pyrazole-5-yl)-4-nitrobenzamide (VU-29) (donated by the Department of Synthesis and Chemical Technology of Pharmaceutical Substances with Computer Modelling Lab, Medical University, Lublin, Poland) was dissolved in a vehicle consisting of 10% Tween-80 (Sigma-Aldrich, Saint Louis, MO, USA) in saline and given at the dose of 30 mg/kg, i.p., in a volume of 1 mL/kg. Injection timepoint and the dose for VU-29 were chosen based on our previous study [53] and preliminary nano LC-MS/MS analysis that showed the presence of this compound in the brain tissues 20 min after i.p. injection (data not published).

### 2.3. Maternal Separation Procedure

MS procedure occurred between (PND)1 to (PND)21 and used 20 dams (10 L for MS stress and 10 L for control). MS practices were based on the protocol of Chocyk et al. [17] with minor modifications. On each of (PND)1–21, from the maternity cages, pups and dams were removed for 180 min (09:00 to 12:00), with the mothers individually placed in holding cages, and each litter positioned in a cardboard container containing fresh bedding material. These containers were subsequently moved to a bigger cage. The dams and pups were returned to the maternity cages post-180 min separation. Control animals were not separated (NS) from their mothers, except during the once-a-week cage cleaning. Male and female pup segregation did not occur during the MS procedure. Post-weaning, at (PND)21, offspring were separated according to sex, housing being at 5 per cage, and were assigned to MRS (male and female rats) and NOR (male animals) on (PND)60.

### 2.4. Spectral Analysis and Quantification of Neurochemicals in the Hippocampus In Vivo

Proton magnetic resonance spectroscopy (^1^HMRS) experiments were performed on a MR 7 T horizontal bore magnet 70/16 PharmaScan, ParaVision 6.0.1 (Bruker BioSpin GmbH, Rheinstetten, Germany) using a volume coil with 72 mm inner diameter for transmission and 20 mm surface loop coil for reception. During this stage of the experiments, the animals were anesthetized with an isoflurane and oxygen mixture (3.5% isoflurane for induction and 1.7–2.2% for maintenance). Respiration rate was monitored throughout scanning and isoflurane concentration was adjusted to maintain respirations within a specified target zone (35–45 rpm). Body temperature was controlled by a rectal thermal probe and maintained at physiological values (about 37 °C) using a warm water circulation system. The number of breaths and body temperature were monitored throughout this part of the study using the MR-compatible Small Animal Monitoring System (SA Instruments, Inc., Stony Brook, NY, USA). Rats were scanned in sessions of ~2.5 h each. Four groups of Wistar rats were examined in the study: separated females, non-separated females, separated males and non-separated males.

### 2.5. Magnetic Resonance Spectroscopy

Three-plane T2-weighted rapid acquisition with relaxation enhancement (RARE) images were acquired before voxel positioning for MRS (TR/TE  =  2500/33 ms, matrix  =  256 × 192, slice thickness  =  0.8 mm, rare factor  =  8, averages  =  1). Using high-quality structural brain images, a volume of interest (VOI) was placed in the right hippocampus, with a size of VOI  =  1.8 mm × 2.7 mm × 4.5 mm (21.9 μL). Magnetic field shimming procedure was performed by employing the built-in Paravision MAPSHIM routine (Bruker BioSpec, Ettlingen, Germany), full width at half maximum was typically in the range of 7.3 to 10 Hz. Proton MRS spectra were acquired over the volume of interest (VOI) using a point resolved spectroscopy (PRESS) sequence (bandwidth 4 kHz, 2048 complex data points, TR 2.5 s, TE 16 ms (TE1/TE2 = 8.43/7.57 ms), 1024 averages, scan time 42 min). The water signal was suppressed by variable RF pulses with optimized relaxations delay (VAPOR). Attenuation of the first RF pulse in VAPOR was selected manually for each animal to reach a satisfactory level of water suppression.

Spectra were processed using LC Model software (Version 6.3-1), which functions fully automatically to decompose an in vivo spectrum into a linear combination of model spectra provided as prior knowledge. In this study, LC Model TM was used in the standard configuration with the analyzing window from 0.2 to 4.2 ppm. The basis set based on the parameters of MRS acquisition (TE =16 ms) was obtained thanks to the courtesy of the LC Model TM software developers and was further employed for quantification. The unsuppressed water signal was used to normalize the fitted signals of metabolites to the water content of the tissue and to calculate absolute concentrations of metabolites in tissue (expressed in millimoles). The Cramér–Rao lower bounds (CRLB), indicating the lower limit of statistical error of the fitted parameters for each metabolite, were determined by LC Model TM. Metabolites with lower than 20% CRLB values are considered as reliable and have been reported in the present study. Concentrations of metabolites are expressed in units mmol/kg tissue, according to the method described in our previous study [54].

### 2.6. Sequence Used for Volumetry Analysis

T2-weighted images for the segmentation portion of this study were acquired in the axial plane spanning the entire brain (image acquisition time ~43 min). Parameters included: repetition time (TR) = 8626.171 ms, echo time (TE) = 50 ms, slice thickness = 0.5 mm, averages = 12, matrix size = 256 × 256 and field of view (FOV) = 30 × 30 mm (spatial resolution = 0.117 × 0.117 mm/pixel).

### 2.7. Volumetry Analysis

Anatomical structures volume analysis was performed according to the following pipeline: (1) Brain extraction and skull stripping—Brain Suite Software, v. 21a (Brain Surface Extractor tool); (2) Nonuniformity correction—correction for image intensity bias (Brain Suite Software, v. 21a); (3) Linear registration to a template—implementation of the same coordinate system for subjects and template images using a FLIRT toolbox for FSL (FMRIB Software Library); (4) Brain tissue segmentation for white matter, grey matter, cerebral spinal fluid was performed on registered images using SPM8; (5) Anatomical structures labelling according to SIGMA rat brain atlas using IBASPM toolbox for SPM8; (6) Brain divided for 246 structures (123 per hemisphere); (7) Volumetric measurements were calculated using IBASPM toolbox for SPM8 by calculating the number of voxels belonging to a given label and multiplying them by the voxel size.

### 2.8. Novel Object Recognition (NOR)

**Figure 1:**
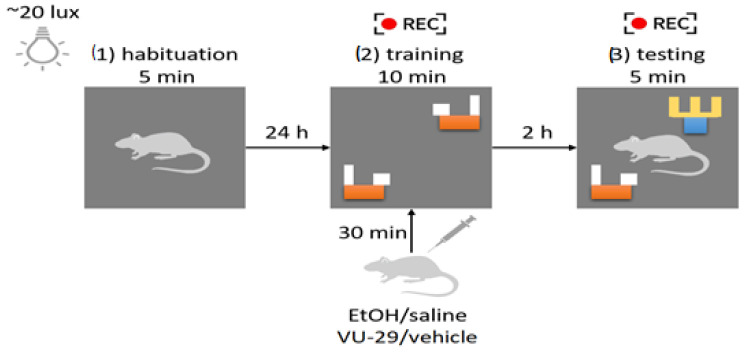
The schedule of the NOR procedure. The procedure consisted of 3 sessions: (1) habituation, (2) training, and (3) testing. Only the first 5 min from the training session were recorded and included in the further analysis.

The experiment was conducted in young adult ((PND)60, N = 48) male Wistar rats, which were divided into 8 groups (6 separated and 2 non-separated) of 6 animals per group. This NOR task was carried out in the same Plexiglas box (40 × 40 × 40 cm) illuminated with ~20 lux light. Animals were placed in the experimental room for 30 min before every session of the NOR task. The procedure included 3 sessions, i.e., (1) habituation followed by the next day (2) the training session and (3) the testing session with 2 h time interval. Two identical objects were placed in diagonal corners of the box during the training session. One of the objects was then replaced by a novel object different in color and shape. Each animal was separately placed in the center of the box facing one of the remaining empty corners. Each animal received two i.p. injections with an interval of 30 min before the training session. Both the training and the testing session were recorded to provide further analysis of animal behaviors. Only the first 5 min of the training session was included in the analysis. Object recognition was manually scored by a blind experimenter, and calculated as the percentage value. All animals reached at least 20 s of total exploration time. The set of objects was chosen based on the preliminary studies, which showed no innate preference between selected objects. After each session of the NOR task, the animals returned to their home cages. The box and objects were thoroughly cleaned with water after each trial, and the order of the treatments was randomized [55,56]. During the training and testing sessions, the total distance travelled (cm) was calculated using EthoVision XT (Noldus, Wageningen, Netherlands). The scheme of the NOR procedure has been provided in Figure 1.

### 2.9. Statistical Analysis

Prism v. 8.0.0 for Windows (GraphPad Software, San Diego, CA, USA) was applied for statistical analysis. The drug effects from behavioral and MRS testing was analyzed by applying two-way analysis of variance (ANOVA) with repeated measures, followed by Tukey’s post-hoc test. Presentation of results were as means ± standard errors of means (SEM) of values. A *p* value less than 0.05 was considered statistically significant for all tests.

## 3. Results

### 3.1. The Effect of MS on the Volume of Adult Rat Brain Structures Important for Recognition Memory

**Figure 2:**
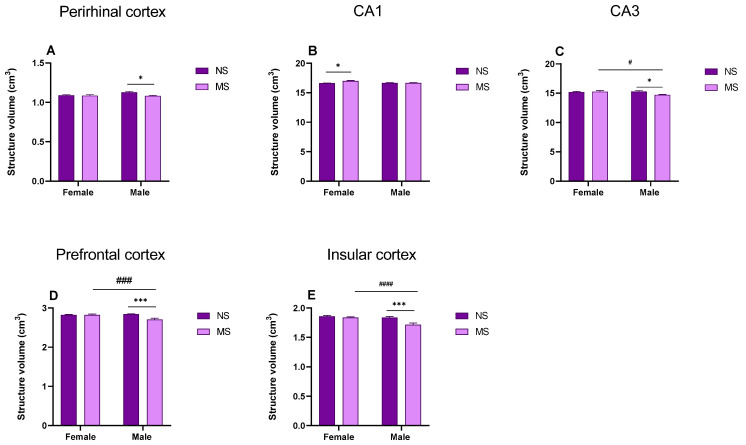
The effect of MS on the volume of adult rat brain structures. (**A**) Perirhinal cortex, (**B**) hippocampal CA1 area, (**C**) hippocampal CA3 area, (**D**) prefrontal cortex, (**E**) insular cortex. Data are presented as mean ± SEM (N = 12/group). * *p* < 0.05; *** *p* < 0.001 vs. control NS, # *p* < 0.05; ### *p* < 0.001; #### *p* < 0.0001 vs. sex. MS, NS-non-stressed.

**Perirhinal cortex**: MRS data indicate that MS caused a statistically significant loss of perirhinal cortex volume in adult males. Two-way ANOVA showed no significant effect of sex of rats [F (1, 44) = 3.534; *p* > 0.05], however, it showed a significant impact of MS [F (1, 44) = 6.874; *p* < 0.05] and interactions of these factors [F (1, 44) = 4.530; *p* < 0.05]. Tukey’s post-hoc test showed that MS males ((PND)1–21) had a reduced volume of the perirhinal cortex compared to NS animals (*p* < 0.05) (Figure 2A).

**Hippocampal CA1 area**: MRS data indicate that MS caused statistically significant increase in hippocampal CA1 (cornu ammonis 1, CA1) volume in adult females. Two-way ANOVA showed significant effect of sex of rats [F (1, 44) = 4875; *p* < 0.05], significant impact of MS [F (1, 44) = 9.816; *p* < 0.01] and interactions between factors [F (1, 44) = 9.702; *p* < 0.01]. Tukey’s post-hoc test showed that MS females ((PND)1–21) have increased hippocampal CA1 volume compared to unstressed animals (*p* < 0.05) (Figure 2B).

**Hippocampal CA3 area**: MRS data indicate that MS results in a statistically significant reduction in hippocampal CA3 volume (cornu ammonis 3, CA3) in adult males. Two-way ANOVA showed no significant effect regarding sex of rats [F (1, 44) = 2939; *p* < 0.05] and MS [F (1, 44) = 2.939; *p* > 0.05], but showed significant impact of interaction of these factors [F (1, 44) = 5.821; *p* < 0.05]. Tukey’s post-hoc test showed that MS males ((PND)1–21) have reduced hippocampal CA3 volume compared to NS males (*p* < 0.05) and MS females (*p* < 0.05) (Figure 2C).

**Prefrontal cortex**: MRS data indicate that MS caused a statistically significant reduction in prefrontal cortex volume in adult males. Two-way ANOVA showed the significant effect of sex of rats [F (1, 44) = 6.043; *p* < 0.05], MS [F (1, 44) = 10.88; *p* < 0.01] and interactions of these factors [F (1, 44) = 11.64; *p* < 0.01]. Tukey’s post-hoc test showed that MS males ((PND)1–21) had reduced prefrontal cortex volume compared to NS males (*p* < 0.001) and MS females (*p* < 0.001) (Figure 2D).

**Insular cortex**: MRS data indicate that MS stress caused a statistically significant reduction in the volume of the insular cortex in adult males. Two-way ANOVA showed a significant effect of sex of rats [F (1, 44) = 12.72; *p* < 0.001], MS [F (1, 44) = 12.33; *p* < 0.01] and interactions of these factors [F (1, 44) = 11.64; *p* < 0.05]. Tukey’s post-hoc test showed that maternally MS males had reduced insular cortex volume compared to NS males (*p* < 0.001) and MS females (*p* < 0.001) (Figure 2E).

### 3.2. Impact of MS on the Level of Amino Acids in the Hippocampus of Adult Rats

**Figure 3:**
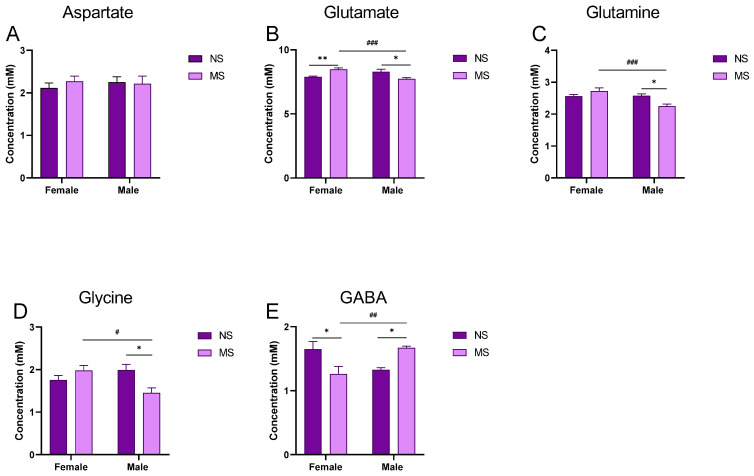
Impact of MS on amino acids level in the hippocampus of adult rats. (**A**) Aspartate, (**B**) glutamate, (**C**) glutamine, (**D**) glycine, (**E**) GABA. Data are presented as mean ± SEM (N = 12/group). * *p* < 0.05; ** *p* < 0.01 vs. control NS, # *p* < 0.05; ## *p* < 0.01; ### *p* < 0.001 vs. sex. MS, NS-non-stressed. Spectra samples are attached to Supplementary Materials (Figures S1 and S2).

**Aspartate**: Two-way ANOVA with repeated measures did not show significant impact of sex of rats [F (1, 44) = 0.08539; *p* > 0.05], MS [F (1, 44) = 0.1950; *p* > 0.05] nor interaction of these factors [F (1, 44) = 0.4742; *p* > 0.05] on the level of aspartate in the hippocampus of adult rats (Figure 3A).

**Glu**: Two-way ANOVA with repeated measures did not show significant impact of sex of rats [F (1, 44) = 1.663; *p* > 0.05] nor MS [F (1, 44) = 0.01721; *p* > 0.05], however, it indicated the significant impact of the interaction of these factors [F (1, 44) = 20.84; *p* < 0.001]. The Tukey post-hoc test showed statistically significant differences between NS and MS males (*p* < 0.05); females (*p* < 0.01); and, between MS males and females (*p* < 0.001) (Figure 3B).

**Gln**: Two-way ANOVA with repeated measures showed statistically significant impact of sex of rats [F (1, 44) = 10.61; *p* <0.01], but not MS [F (1, 44) = 1.378; *p* > 0.05], however, it did indicated significance in the interaction of these factors [F (1, 44) = 11.82; *p* <0.01]. Comparisons between the groups with post-hoc Tukey test indicated statistically significant differences between NS and MS males (*p* < 0.05) and between MS males and females (*p* < 0.001) (Figure 3C).

**Glycine**: Two-way ANOVA with repeated measures did not show significant impact of sex of rats [F (1, 44) = 1.522; *p* > 0.05] or MS [F (1, 44) = 1.838; *p* > 0.05], however, it indicated a significant impact of the interaction of these factors [F (1, 44) = 10.54; *p* < 0.01]. The Tukey post-hoc test showed statistically significant differences between NS and MS males (*p* < 0.05) and between MS males and females (Figure 3D).

**GABA**: Two-way ANOVA with repeated measures did not show significant impact of sex of rats [F (1, 44) = 0.2446; *p* > 0.05] nor MS [F (1, 44) = 0.06737; *p* > 0.05], however, it indicated a significant impact of interaction of these factors [F (1, 44) = 17.86; *p* < 0.001]. The Tukey post-hoc test showed statistically significant differences between NS and MS males (*p* < 0.05); females (*p* < 0.05); and, between MS males and females (*p* < 0.01) (Figure 3E).

### 3.3. Impact of Ethanol on Memory in the NOR Test in Adult Rats Exposed to Repeated MS; Impact of VU-29 on Ethanol-Impaired Memory in MS Rats

**Figure 4:**
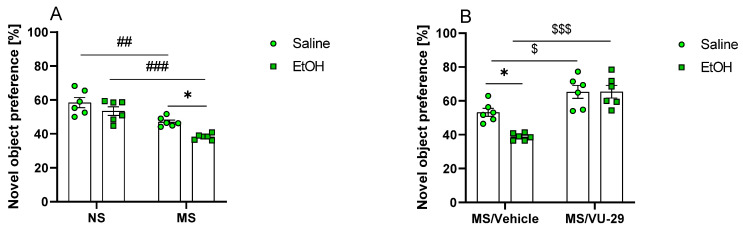
Influence of MS on recognition memory and impact of ethanol on MS-induced deficit in recognition memory in adult male rats ((PND)60). Influence of VU-29 on memory deficits in adult male rats exposed to repeated MS during adolescence. Two separate analysis was performed to determine (**A**) the effect of MS and ethanol on novel object preference, (**B**) the effect of VU-29 and ethanol on novel object preference in rat offspring subjected to MS. Training-testing interval was 2 h. Data are presented as mean ± SEM (N = 6/group). * *p* < 0.05 vs. EtOH MS, ## *p* < 0.01; ### *p* < 0.001 vs. NS, $ *p* < 0.05; $$$ *p* < 0.001 vs. VU-29. EtOH-ethanol, MS-maternal separation, NS-non-stressed.

A two-way ANOVA was performed to analyse the effect of MS and ethanol on novel object preference (treatment groups included in the analysis: Control NS, EtOH NS, Control MS, EtOH MS). Our work revealed that there was no statistically significant interaction between the effects of MS and ethanol [F (1, 20) = 0.8808, *p* > 0.05] nor a significant main effect of ethanol [F (1, 20) = 2.009, *p* = 0.1718]. However, ANOVA analysis showed that MS [F (1, 20) = 41.47, *p* < 0.001] and ethanol administration [F (1, 20) = 11.15, *p* < 0.01] had a statistically significant effect on novel object preference. Post hoc analysis showed that there was no significant difference in object preference in either NS adults. Nonetheless, Tukey’s multiple comparisons test revealed that both Control MS and EtOH MS groups (*p* < 0.01 and *p* < 0.001, respectively) expressed a significantly decreased novel object preference when compared to corresponding non-MS groups. Furthermore, there was significant difference between ethanol-treated and control MS rats (*p* < 0.05) (Figure 4A).

The second two-way ANOVA was performed to determine the effect of VU and ethanol on novel object preference in adult rats subjected to MS (treatment groups included in the analysis: Control MS, EtOH MS, VU-29 MS, VU + EtOH MS). Our work demonstrated that there was a statistically significant interaction between the effects (VU-29 pretreatment x EtOH treatment) [F (1, 20) = 6.158, *p* < 0.05]. Simple main effects analysis showed that ethanol significantly decreased novel object preference [F (1, 20) = 5.954, *p* < 0.05]. Moreover, the analysis revealed that VU-29 had a statistically significant effect on the outcome of the NOR test [F (1, 20) = 43.28, *p* < 0.001]. Importantly, the post hoc analysis revealed that rats pretreated with VU-29 before ethanol administration had a higher preference for the novel object when compared to the EtOH group (*p* < 0.001). Moreover, VU-29 ameliorated memory deficits induced by MS control group (*p* < 0.05) (Figure 4B).

### 3.4. Locomotor Activity in NOR Task

During the training session, a two-way ANOVA was performed to analyse the effect of MS and ethanol on locomotor activity (treatment groups included in the analysis: Control NS, EtOH NS, Control MS, EtOH MS). It was revealed that there was no statistically significant interaction between the effects of MS and ethanol [F (1, 20) = 0.08608, *p* > 0.05] and no significant main effect of ethanol [F (1, 20) = 2.255, *p* > 0.05] nor MS [F (1, 20) = 2.73, *p* > 0.05] (Table 1).

The second two-way ANOVA was performed to determine the effect of VU and ethanol on locomotor activity in rat offspring subjected to MS (treatment groups included in the analysis: Control MS, EtOH MS, VU-29 MS, VU + EtOH MS). Our work revealed that there was no statistically significant interaction between the effects (VU-29 pretreatment x EtOH treatment) [F (1, 20) = 4.258, *p* > 0.05]. Simple main effects analysis showed that neither ethanol [F (1, 20) = 0.02543, *p* > 0.05] nor VU-29 had statistically significant effect on the locomotor activity [F (1, 20) = 1.396, *p* > 0.05] (Table 1).

During the testing session, a two-way ANOVA was performed to analyse the effect of MS and ethanol on locomotor activity (treatment groups included in the analysis: Control NS, EtOH NS, Control MS, EtOH MS). It was revealed that there was no statistically significant interaction between the effects of MS and ethanol [F (1, 20) = 0.4622, *p* > 0.05] and no significant main effect of ethanol [F (1, 20) = 0.2093, *p* > 0.05] nor MS [F (1, 20) = 2.370, *p* > 0.05] (Table 1).

The second two-way ANOVA was performed to determine the effect of VU and ethanol on locomotor activity in rat offspring subjected to MS (treatment groups included in the analysis: Control MS, EtOH MS, VU-29 MS, VU + EtOH MS). Our work revealed that there was no statistically significant interaction between the effects (VU-29 pretreatment × EtOH treatment) [F (1, 20) = 0.0022, *p* > 0.05]. Simple main effects analysis showed that neither ethanol [F (1, 20) = 0.04073, *p* > 0.05] nor VU-29 had statistically significant effect on the locomotor activity [F (1, 20) = 0.5240, *p* > 0.05] (Table 1).

## 4. Discussion

The result of the present study (using ^1^HMRS) indicated that young adult male rats ((PND)60) exposed to MS ((PND)1–21, 180 min/day) show loss in volume of the brain structures relevant for recognition memory (the perirhinal cortex, prefrontal cortex and insular cortex and the CA3 region of the hippocampus). Furthermore, in the hippocampus, deficits in the level of Glu and related amino acids (Gln and glycine) and increases in GABA levels were observed. In young adult female rats exposed to MS, of the brain structures, only the values of CA1 region of the hippocampus were increased. In the hippocampus, in contrast to the males, the glutamate level was increased, while GABA level was decreased and no significant changes were observed in the levels of aspartate, Gln and glycine. Additionally, male rats exposed to MS displayed deficits in retrieval of recognition memory. This memory deficit was ameliorated by VU-29, the mGlu5 receptor PAM. Finally, we noted that acute ethanol administration potentiated memory impairment in adult male rats with MS, while VU-29 pretreatment reversed this deficit.

Published data show that early MS did not affect the volume of selected brain regions (prefrontal cortex, dorsal striatum, and dorsal and ventral hippocampus) in male 90-day-old rats as measured by MRI [57]. Moreover, a previous MRI study reported that MS did not change hippocampal volume in 70-day-old male rats [58]. Another MRI study also reported reduced hippocampal values after MS in young mice (both sexes), but it was normalized in adulthood (70 days) and hippocampal neurogenesis was also unaffected, although, hippocampal synaptic plasticity assessed by long-term potentiation (LTP) was impaired in adult mice after MS [59]. Conversely, our study in young adult ((PND)60) male rats shows that repeated MS during adolescence ((PND)1–21, 180 min/d) reduced the volume of the perirhinal cortex, prefrontal cortex, insular cortex and the CA3 region of the hippocampus—the brain structures required for generation of recognition memory [60]. However, such changes were not observed in female rats, apart from the CA1. It seems that the above shown differential outcomes produced by MS may depend on the separation paradigm, time point examined, and animal species used, suggesting that the precise mechanisms underlying these modifications need further examination. Of note, several studies in humans showed that early life stress (caused by childhood emotional or sexual maltreatment) is associated with decreased volume of the prefrontal cortex and/or the hippocampus during adulthood [61,62,63].

The changes in brain volume can be due to structural reorganization of brain regions, involving changes in neurons and glial cells [64,65]. The effects of postnatal stress on neurogenesis have been widely studied in rodents. Most of the studies show a trend of a decreased proliferation and/or a decreased cell survival in the dentate gyrus (DG) of male and female rodents immediately after MS stress exposure. However, in adult males, initial changes are followed by an increase or decrease in these parameters [66,67,68,69]. In females, however, early effect of stress on neurogenesis subsides in adulthood [70]. Thus, the alteration in development of the hippocampus induces permanent changes in hippocampal function and disrupts the normal concentrations of important neurotransmitters, which are accompanied by behavioral deficits mainly in males [71].

In our MRS study, MS induced changes were found in the level of the excitatory and inhibitory amino acids in the hippocampus and were more pronounced in the adult males. In these animals, we observed deficits in Glu, Gln and glycine levels and, conversely, GABA level increase. According to published data, the lower levels of Glu and Gln could be due to decreases in the number of hippocampal astrocytes in rats [72] exposed to MS. In astrocytes (astroglia)**,** Glu is converted into Gln and then recycled back to glutamatergic neurons (glutamate-glutamine cycle) [73]. According to the neuron-glia integrity theory, the Glu-Gln-GABA cycle functions as the neuro-chemical substrates of the neuron-astrocyte entity. Changes in Glu, Gln and/or GABA in a brain region are, thus indicative of an imbalanced Glu-Gln-GABA cycle or an impaired neuron-astrocyte entity.

Furthermore, published studies have shown that male adult offspring of stressed mothers exhibit higher levels of ionotropic and metabotropic glutamate receptors than do control rats. These offspring also show long-lasting astroglia hypertrophy and a reduced dendric arborization with synaptic loss [74]. These results allow us to hypothesize that the variations seen in the MS rats might be a compensatory neuroprotective process against glutamatergic hyperactivity and excitotoxicity. The specificity of the changes in the hippocampus could reflect impaired glutamatergic function in an area which could be the location for memory and learning deficits of MS rat models [75,76,77].

In our study, most of the changes observed in male adult rats exposed to MS in adolescence were not detected in the female counterparts. Thus, we showed significant gender-specific differences in the hippocampal amino acid levels, such as Glu, Gln, glycine and GABA. In adult female rats exposed to MS, the glutamate levels were increased, while GABA levels were decreased in the hippocampus with no significant changes in the level of aspartate, Gln and glycine. Few studies report prenatal stress consequences in both sexes, and the majority agree that learning deficits, LTP and dendric density reductions (among others) are seen mainly in males, while females are susceptible to anxiety, depression and response to changes in the hypothalamic-pituitary-adrenal axis [78,79]. Indeed, in our study, the level of GABA was decreased in the hippocampus of female rats, suggesting an anxiety-like behavior in adult females exposed to MS stress during adolescence. Moreover, Bowman et al. [80] reported gender differences in MS offspring in the hippocampal and prefrontal cortex concentration of monoamines (noradrenaline, serotonin and dopamine). It is also recognized that estrogens can increase spines, glutamate receptor binding and LTP in the hippocampus [81]. This could explain the gender specific response to MS and the female capability to overcome insults received during brain development.

Regarding N-acetyl-aspartate, this is considered the neurochemical correlate of the axon-myelin entity because it is involved in myelination and axon-glia signaling, in addition to playing a role in osmoregulation [82,83]. Chronic administration of D-Aspartate has been proposed as therapeutic treatment in diseases related to myelin dysfunction and NMDA receptors hypofunction, including cognitive deficits [84]. Our study showed that aspartate level was not significantly changed in the hippocampus of MS rats relative to control group in both sexes, suggesting that myelination integrity was not impacted in this brain structure, although such changes were observed by others in the medial prefrontal cortex (mPFC) [85]. Future studies using other complementary methods will be undertaken to support our (in vivo ^1^HMRS) results concerning the level of metabolites in the hippocampus in rats with MS.

Glutamatergic neurotransmission is critical, not only for the formation of object recognition memories, but also for the discrimination of novel from familiar objects, and the hippocampus is engaged in this process [34,86]. As shown in the experimental data of several studies, hippocampal activity is increased significantly during test sessions, including both hippocampal glutamate efflux and mean firing rates of CA1 neurons [87,88,89]. Consistent with the data on hippocampal synaptic signaling and plasticity, our behavioral studies indicate that MS is associated with a significant decrease in the cognitive performance in male rats and we noted that MS impaired the preference for novelty in males, as shown in the NOR task. In line with our data, a vast number of experimental and clinical evidence confirms that early-life stress, including that associated with MS, might exert deleterious effects on brain structure and function later in life [22,45,90,91,92]. Herein, we hypothesize that these cognitive declines in male rats may be the results of deficits in glutamate neurotransmission in the brain structures connected with recognition memory, as we showed above. Considering this hypothesis, we did not perform the NOR task in female rats, and this is a limitation of our study and needs future support/investigation.

Published data provide evidence concerning NMDA mechanisms related to recognition memory processes and show that recognition memory for objects, places or associations between object and places depends on NMDA neurotransmission within the perirhinal cortex, hippocampus and medial prefrontal cortex [93]. Furthermore, NMDA receptor activation has been shown to be necessary for the most common forms of LTP and long-term depression (LTD) in the hippocampus [94,95]. Thus, we suggest that the lower level of glutamate at the NMDA receptor, and that of glycine as modulator of the NMDA receptor [96] in the hippocampus, were responsible for deficits in memory retrieval in the NOR task in our study.

Our findings also demonstrated that MS-induced recognition memory deficits in male rats were potentiated by acute administration of ethanol at doses that did not induce memory impairment. These data clearly indicate, for the first time, that early exposure to MS increases susceptibility to acute effects of ethanol during adulthood. Ethanol is a modulator of learning-related synaptic plasticity [97]. Several studies show that ethanol potently inhibits NMDA receptors [49]. Thus, we suggest that ethanol potentiated MS-induced deficits in glutamate signaling in the hippocampus responsible for memory processes (LTP and LTD). Our previous data showed that NMDA receptors are engaged in MS induced increase of ethanol drinking in adolescent/adult rats and spatial learning deficits. These effects were reversed by potentiation of glutamatergic neurotransmission by Org 24598, a glycine transporter 1 (GlyT1) inhibitor [50]. The present study shows that MS lowered the level of glycine in the hippocampus of adult male rats. According to the distribution of glycine transporters (GlyT1/GlyT2) in the hippocampus, it has been demonstrated that glycine is mainly accumulated in astrocytes, and hence astrocytes rather than neurons are the main source of hippocampal glycine [98,99]. Because astrocyte-derived glycine, by modulating the level of NMDA receptor activation, may regulate the extent of LTP/LTD synaptic activity [98,100,101], our data suggest the crucial role of the NMDA/glycine receptor in memory deficits induced by MS and those potentiated by ethanol.

mGlu5 receptors are physically connected with NMDA receptors and they interact with, and modulate the function of one another in several brain regions, including the hippocampus [102]. mGluR5 plays an important role in both LTP and LTD, suggesting that mGluR5 PAMs may also have utility in improving impaired cognitive function. VU-29, a mGlu5 receptor PAM, in previous study, ameliorated many ethanol effects, including memory deficits induced by ethanol [53,103]. In the present experiments, this compound reversed recognition memory deficits induced by MS alone, as well as those induced by MS and potentiated by ethanol. Thus, it is possible that potentiation of synoptically activated mGlu5 receptors by VU-29 enhances the induction of normal NMDA receptor-dependent LTP, as suggested by other authors [104] and reverses/protects the memory deficits induced by MS or this MS-induced deficit potentiated by ethanol. Further MRS study are needed to confirm the impact of VU-29 on the amino acid, especially glutamate, levels in the brain areas affected by MS and ethanol.

Taken together, our study indicated that MS stress during adolescence has sex-specific impact on the volume of the brain structures connected with recognition memory in adult rats and the level of amino acids in the hippocampus. Deficits were more pronounced in male rats, and they have deleterious impact on recognition memory. Enhancement of glutamatergic neurotransmission via the mGlu5 receptor PAM reversed cognitive deficits. Thus, compounds targeting mGlu5 receptors, specifically subtype-selective PAM, could provide a compelling alternative approach to fill the unmet clinical needs for patients with cognitive deficits induced by early life stress, such as MS.

## Figures and Tables

**Table 1:** Locomotor activity in the NOR task.

Treatment Group	Total Distance Traveled (cm)	
	Training (Mean ± SEM)	Testing (Mean ± SEM)
Control NS	1390 ± 133.5	1662 ± 121.0
EtOH NS	1228 ± 144.1	1533 ± 109.1
Control MS	1652 ± 74.54	1760 ± 75.69
EtOH MS	1410 ± 168.1	1786 ± 140.1
VU MS	1240 ± 55.86	1871 ± 190.6
VU + EtOH MS	1522 ± 165.6	1912 + 213.4

## Data Availability

Data sharing is not applicable to this article.

## Supplementary Materials

The following supporting information can be downloaded at: https://www.mdpi.com/article/10.3390/biom13101449/s1, Figure S1. In vivo protein magnetic resonance spectrometry (1HMRS) spectrum in the hippocampus of one randomly chosen adult male rat: (A) the control group, (B) the group that received ethanol between (PND)4-9. 7T Bruker, animal system. Glutamate (Glu), glutamine (Gln), glycine (Glc), gamma-aminobutyric acid (GABA); Figure S2. In vivo protein magnetic resonance spectrometry (1HMRS) spectrum in the hippocampus of one randomly chosen adult female rat: (A) The control group, (B) The group that received ethanol between (PND)4-9. 7T Bruker, animal system. Glutamate (Glu), glutamine (Gln), glycine (Glc), gamma-aminobutyric acid (GABA).
