# Supplementary material for: Neonatal Maternal Separation Induces Sexual Dimorphism in Brain Development: The Influence on Amino Acid Levels and Cognitive Disorders
Source: Biomolecules. 2023 Sep 26;13(10):1449. doi: 10.3390/biom13101449 (PMC10605115; doi:10.3390/biom13101449)
Supplement: Supplementary file 1 [file biomolecules-13-01449-s001.zip › biomolecules-2558508-supplementary.pdf]

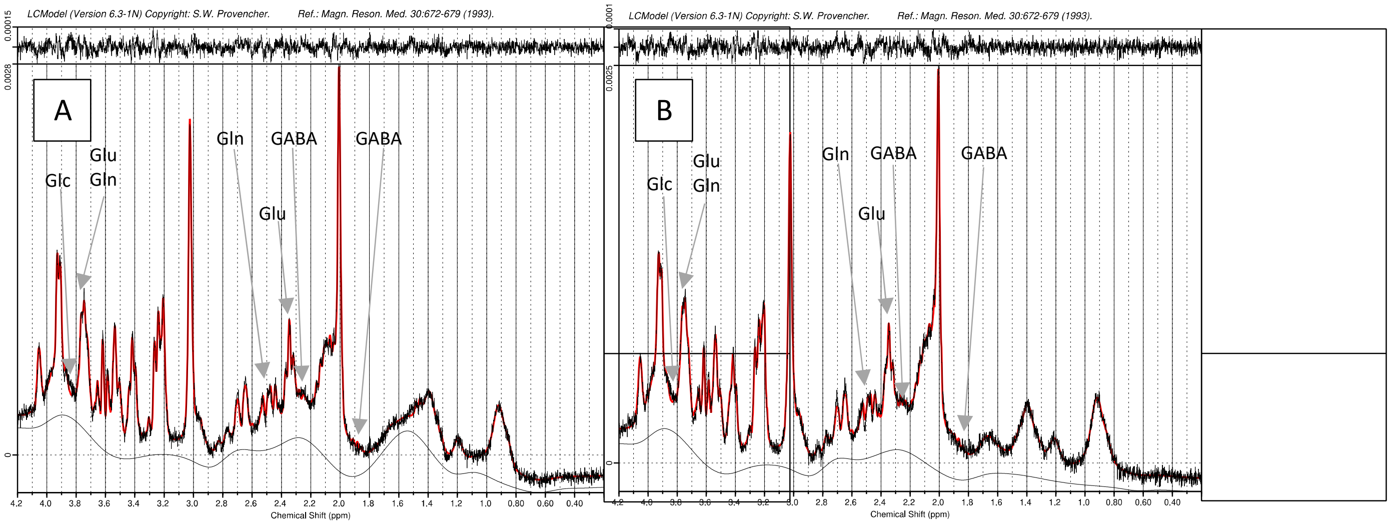

Figure S1. *In vivo* protein magnetic resonance spectrometry (^{1}HMRS) spectrum in the hippocampus of one randomly chosen adult male rat: (**A**) the control group, (**B**) the group that received ethanol between PND4-9. 7T Bruker, animal system. Glutamate (Glu), glutamine (Gln), glycine (Glc), gamma-aminobutyric acid (GABA).

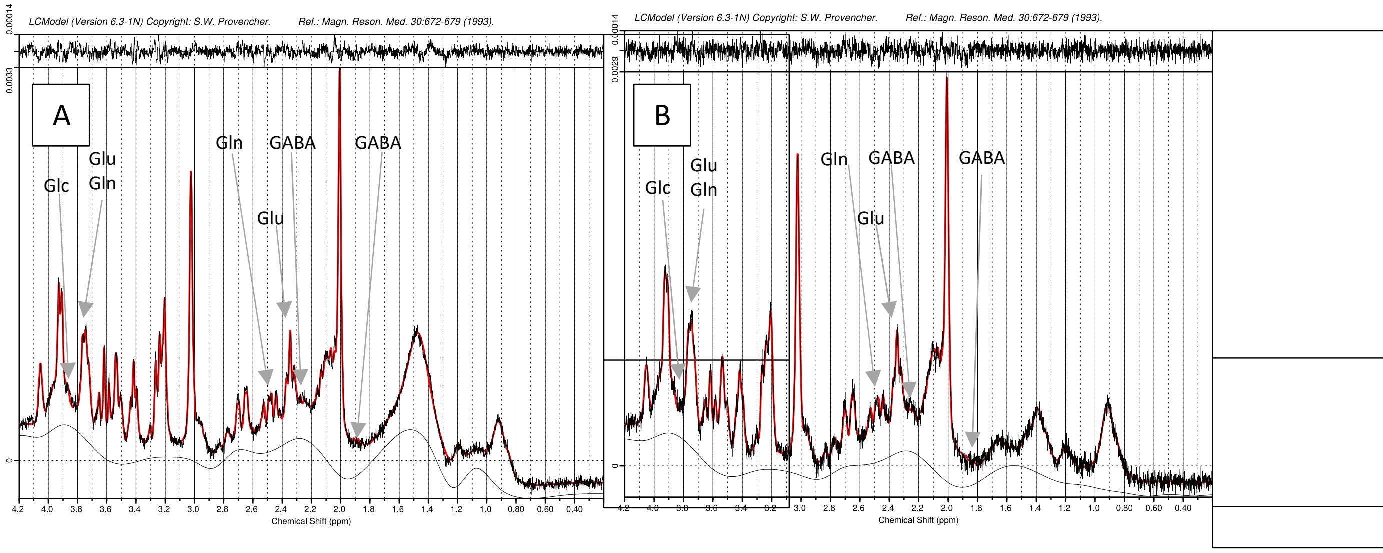

Figure S2. *In vivo* protein magnetic resonance spectrometry (1HMRS) spectrum in the hippocampus of one randomly chosen adult female rat: (**A**) The control group, (**B**) The group that received ethanol between PND4-9. 7T Bruker, animal system. Glutamate (Glu), glutamine (Gln), glycine (Glc), gamma-aminobutyric acid (GABA).
